# Seasonal Changes in Colour: A Comparison of Structural, Melanin- and Carotenoid-Based Plumage Colours

**DOI:** 10.1371/journal.pone.0011582

**Published:** 2010-07-14

**Authors:** Kaspar Delhey, Claudia Burger, Wolfgang Fiedler, Anne Peters

**Affiliations:** 1 Behavioural Ecology of Sexual Signals Group, Vogelwarte Radolfzell, Max Planck Institute for Ornithology, Radolfzell, Germany; 2 Vogelwarte Radolfzell, Max Planck Institute for Ornithology, Radolfzell, Germany; University of Sussex, United Kingdom

## Abstract

**Background:**

Plumage coloration is important for bird communication, most notably in sexual signalling. Colour is often considered a good quality indicator, and the expression of exaggerated colours may depend on individual condition during moult. After moult, plumage coloration has been deemed fixed due to the fact that feathers are dead structures. Still, many plumage colours change after moult, although whether this affects signalling has not been sufficiently assessed.

**Methodology/Principal Findings:**

We studied changes in coloration after moult in four passerine birds (robin, *Erithacus rubecula*; blackbird, *Turdus merula*; blue tit, *Cyanistes caeruleus*; and great tit, *Parus major*) displaying various coloration types (melanin-, carotenoid-based and structural). Birds were caught regularly during three years to measure plumage reflectance. We used models of avian colour vision to derive two variables, one describing chromatic and the other achromatic variation over the year that can be compared in magnitude among different colour types. All studied plumage patches but one (yellow breast of the blue tit) showed significant chromatic changes over the year, although these were smaller than for a typical dynamic trait (bill colour). Overall, structural colours showed a reduction in relative reflectance at shorter wavelengths, carotenoid-based colours the opposite pattern, while no general pattern was found for melanin-based colours. Achromatic changes were also common, but there were no consistent patterns of change for the different types of colours.

**Conclusions/Significance:**

Changes of plumage coloration independent of moult are probably widespread; they should be perceivable by birds and have the potential to affect colour signalling.

## Introduction

Plumage coloration is a prominent aspect of avian visual communication, playing important roles in such disparate functions as crypsis, competition and advertisement, whereby striking or contrasting colour patches often act as inter- and intra-sexual signals of condition and individual quality [1]. Since most plumages are produced once per year, plumage colour is generally perceived as a static trait, fixed after the annual moult. However, the “fading” of colours between moults is considered a common phenomenon in many bird species [2] and classifying plumage colours as static traits may be misleading. Indeed, plumage is exposed to a variety of biotic and abiotic factors that could alter coloration. Colour expression could change due to microbial activity [3], ectoparasites [4], accumulation of dirt particles [5], feather abrasion [2], [6]–[8], application of cosmetics [9], [10] or exposure to ultraviolet (UV) light [11], [12]. These effects can in turn be modulated through investment in plumage maintenance [13].

Seasonal changes in plumage coloration are rarely considered of importance for signalling and especially the relevance of signal alteration outside the breeding season has rarely been studied [14], [15]. Compared to the amount of literature on functional aspects of coloration, only relatively few species and colour patches have been examined in detail for seasonal changes so far [16], [17], [5], [18]–[23]. Most of these studies focused on (presumed) signalling colours and revealed, in general, considerable changes in colour characteristics that might affect signalling through plumage coloration.

Plumage colours are produced through a variety of mechanisms, and the importance and extent of annual colour change is likely to vary with the different types of coloration. The commonest pigment-based colours are produced by carotenoids (derived from the diet, producing greenish, yellow, orange and red colours) and melanins (occurring in two forms, grey to black eumelanins and brown to red phaeomelanins [24], [25]). In addition, UV, blue and white structural colours are caused by nano-scale reflective tissues that result in structural interference [26]. Traditionally, the production of carotenoid-based colours is assumed to be highly condition-dependent while melanin-based colours seem to be mainly under genetic control ([27] but see [28]). Carotenoid-coloured feathers appear particularly sensitive to bleaching by (UV) light or abrasion [29], [19], whereas melanins are known to strengthen feather structure [29], [30] and thus could limit colour change if for instance these feathers are less sensitive to abrasion [19]. Structural colours often show high UV reflectance, and UV reflectance has been hypothesised to be particularly sensitive to decline, mainly due to dirt accumulation [5], [13]. Indeed, the one structural colour trait examined throughout the season, the UV/blue crown of the blue tit, (*Cyanistes caeruleus*) shows a clear reduction of UV reflectance with time [5], [22], [31].

Thus, although several studies have documented that plumage colour can change meaningfully after moult, the generality of this phenomenon remains unclear. Moreover, as very different methods to quantify colour variation have been used it is not possible to compare the magnitude of change among different species or colour types or, more importantly, to determine whether seasonal colour differences are perceivable by the birds. Here we systematically investigate the annual pattern of colour change in plumage patches of carotenoid, melanin and structural origin, for males and females of four species of European passerines. Additionally, we compare plumage colour change with seasonal colour changes in a known dynamic trait (bill coloration). To this end we developed a method to quantify variation in bird coloration based on physiological models of avian colour vision. This method allows comparable estimation of perceivable differences in different colours, something that is not often possible using more “traditional” colour variables (see [32] for a review).

## Methods

### Study species

We studied seasonal variation in coloration of four species of European passerine birds, namely robin (*Erithacus rubecula*), blackbird (*Turdus merula*), blue tit (*Cyanistes caeruleus*) and great tit (*Parus major*). These species show a broad range of colorations (structural, melanin- and carotenoid-based) and are resident and common throughout the year in the study area. We caught birds using mist nets in the area of Möggingen (47°45'N, 8°59'E), Germany, at about weekly intervals between April 2005 and January 2008. Captures were part of a constant effort bird banding site established to monitor bird populations in the area. Bird capture and measurement was done under approval from the Regierungspräsidium Freiburg (Aktenzeichen 55/8853.17/0). Mist nets were monitored every hour (more often in cases of inclement weather) and birds processed and released quickly (usually in less than 30 min) after having been removed from the net. Priority was given to females in breeding condition that may have been incubating or brooding. We did not measure birds being still completely or partly in juvenile plumage, as well as adult birds that showed heavy moult (showing more than 20 growing contour feathers). This reduced sample sizes for the months of moult (June-October). All four studied species undergo one single (post-breeding) annual moult [33]. We determined the sex of great tits and blackbirds unambiguously using plumage traits [33]. For blue tits and robins we took small blood samples from the brachial vein and determined sex using molecular markers [34], [35]. We defined ‘year’ as starting on the first of August, which is just after the peak of moult (which was roughly similar for all four species). Thus, month  = 1 corresponds to August and month  = 12 to July.

For each species we obtained samples for most months of the year, which varied somewhat for the different colour patches (not all plumage patches were measured for all birds), resulting in 236 samples for the robin, 192–194 for the blue tit, 299–300 for the great tit and 130–131 for the blackbird. For more details on monthly sample sizes for each species, sex and colour patch see Table S1. During the study period, between one fifth and one third of individuals were caught and measured more than once (robin: mean  = 2.87 captures, range: 2–8; blue tit: mean  = 3.1, range: 2–9; great tit: mean  = 2.73, range: 2–7; blackbird: mean  = 2.83, range: 2–5). However, recaptures were not sufficiently evenly spread within and between moult years to enable within-individual analysis of changes over the year (see also statistical analysis).

Plumage patches that were colour measured in each species were chosen to cover a variety of colours and included patches with an assumed signalling function (for example the crown of the blue tit) as well as presumably non-signalling or cryptic patches (for example the back of the great tit) or patches of unknown function. Plumage patches measured were: robin, breast (red) and back (brown); blackbird, head, breast and back (blackish in males, brownish in females); blue and great tit, head (blue in the blue tit, black in the great tit), cheek (white), breast (yellow) and back (olive-green). We also included one non-plumage colour patch in our analysis, the yellow-orange bill of the blackbird, a known dynamic trait that functions in sexual signalling [36]–[38], but with its seasonal patterns being still unknown. Based on the general colour producing mechanism these colour patches can be roughly divided as: of structural origin (blue tit crown and cheek, great tit cheek), melanin-based (great tit crown, back and breast of the robin and all patches of the blackbird except for the bill) and carotenoid-based (breast and back of blue tit and great tit and bill of the blackbird). It should be noted that this classification only reflects the main colour-producing mechanism. For instance the black crown of the great tit displays a shortwave peak and most likely has a structural component as well, while the olive-green back of blue and great tits is due the deposition of carotenoids on melanised feathers (see Discussion for more details).

### Reflectance spectrometry

Reflectance measurements from 300 to 700 nm (which encompasses the range of visual sensitivity of passerine birds) were made using an Avaspec 2048 spectrometer and an Avalight DH-S Deuterium Halogen light source (for more details see [39]). Five replicate reflectance spectra (in 1-nm steps) were obtained from different but standardized spots for each colour patch and imported into spreadsheets for further processing.

### Quantification of chromatic and achromatic variation in colour

Our goal was to quantify seasonal variation in coloration in such a way that it would be comparable in magnitude among different coloured patches, sexes and species. To achieve this we used models of avian colour vision, which are based on our current knowledge of the physiology of bird eyes and visual systems [40], [41].

Diurnal birds possess four types of single cones that are used for colour vision and are sensitive to very short (VS), short (S), medium (M) or long (L) wavelengths [41]. We used the model proposed by Vorobyev et al. [40], where the sensitivity of the cone types, the reflectance spectrum of the plumage (or bill) and the spectrum of irradiant light are integrated over wavelength to calculate cone quantum catches for each cone type. Birds can roughly be divided in two groups depending on the peak sensitivity of their VS cones: species with U-type or with V-type eyes [42], [43]. All species in this study belonged to the Passerida, which have been shown to have U-type eyes [44], [42]. As our main interest here is to analyse colour changes in the context of intraspecific signalling (as opposed to detectability to predators for example) we used generalized cone sensitivity functions for U-type eyes from Appendix A in Endler and Mielke [43]. As measure for irradiant light we used the spectrum of standard daylight D65, as an earlier study showed that the degree of discriminable variability was mostly unaffected by differences in illuminants [39].

Cone quantum catches were computed for the four different cone types using equation (1) in [40]. Subsequently, we averaged the cone quantum catches for each of the five replicate measurements for each measured colour patch, obtaining one single set of cone quantum catches per patch for each individual.

**Figure 1:**
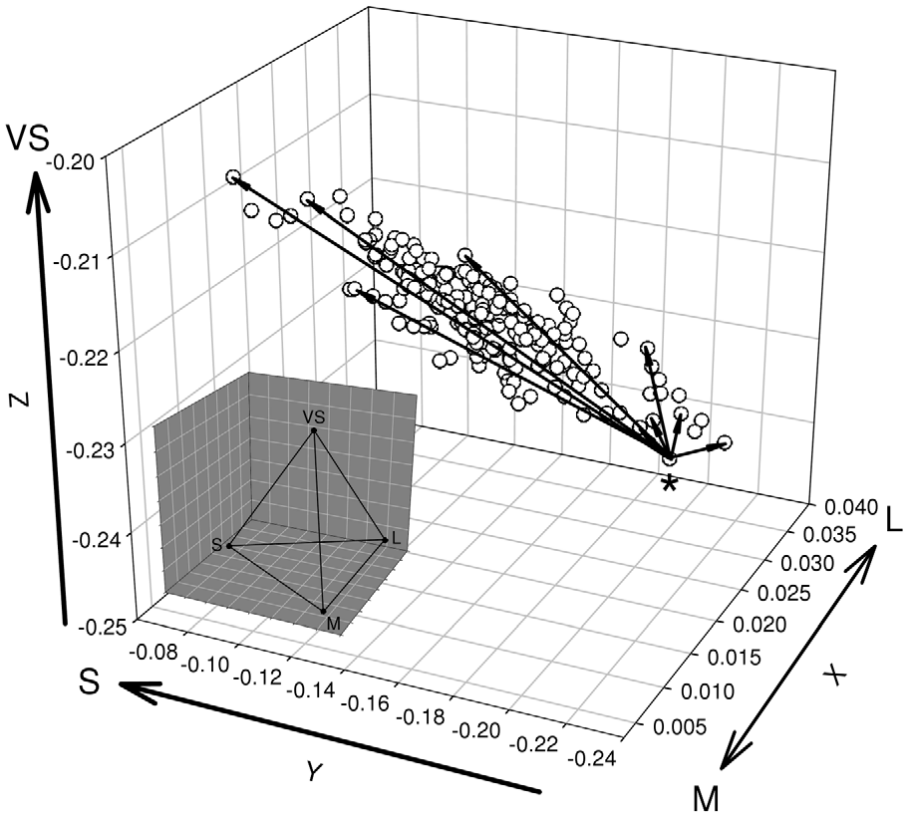
Graphical representation of the procedure used to compute chromatic variation. Principal component analysis of the xyz coordinates reveals that most of the chromatic variation (>80%) can be described by a single principal component (PC1). By selecting the data point with the lowest PC1 value (marked here by the asterisk) and computing discriminability (ΔS) between this point and all other points in the sample (black arrows, only a few arrows are depicted for clarity) we obtain a measurement of chromatic variation that takes into account the different signal-to-noise ratios of the four single cone types in the avian retina and can be directly compared between different colour types. The data represented in the figure corresponds to the yellow breast of the blue tit. Inset shows the position of the vertices of the tetrahedral visual space of birds. In this representation larger values of x represent higher stimulation of the L cone and lower stimulation of the M cone, larger values of y correspond to higher stimulation of the S cone and larger values of z increased stimulation of the VS cone.

Relative cone quantum catches (each cone quantum catch divided by the sum of all four) were transformed into three independent coordinates x, y and z (using equations A9, A10 and A11 from [45]). These three coordinates define the position of each reflectance spectrum in a three-dimensional tetrahedral colour-space where each of the four apexes of the tetrahedron represents the exclusive stimulation of a single cone type (higher x =  higher stimulation of the L cone and lower stimulation of the M cone, higher y  =  higher stimulation of the S cone, higher z  = higher stimulation of the VS cone, Figure 1). In this colour space, different colour patches form more or less discrete clouds of points that generally show one clear axis of variation (Figure 1). The distance between two points in this colour space as computed based on the visual model [40] represents the degree of chromatic difference between them. This model takes the differences in noise levels among cone types into account to calculate chromatic discriminability (ΔS) between two points in the three-dimensional colour space [40]. We computed ΔS using equations 1, 2, 3 and 8 from Vorobyev et al. [40] with a Weber fraction of 0.05 and cone proportions of 1∶1∶2∶2 [43]. For more details see [39]. Values of ΔS are given in units of jnd (just noticeable differences) where in theory differences between two colours <1 jnd should not be discriminable for birds. Thus, ΔS provides us with a metric that is directly comparable among different colours and measured in units that reflect how perceivable differences between colours are to birds. The only problem with ΔS is that it gives an absolute measurement of distance in the avian visual space with no information regarding the direction of this difference. This problem could be circumvented by computing ΔS between each point and a single reference point, ideally at one of the extremes of the cloud of points (Figure 1).

In order to identify this reference point using the same criterion for all colour patches we summarized the information contained in the three coordinates x, y and z by calculating principal components (PCs) using SPSS 15.0. PCs were computed separately for each species and patch using a covariance matrix in order to maintain unequal variation of the coordinates. The first unrotated PC (PC1) explained between 85% and 97% of the variation for each colour patch and represents thus the main direction of colour variation across individuals (see also [46]) confirming that most of the chromatic variation is restricted to only one axis of variation. Correlations of the coordinates with PC1 were negative for x (except for the back of the great tit) and positive for y and z (for details on Principal Component Analyses see Table S2), and thus represents higher stimulation of the shortwave cones (VS and S) relative to the more longwave cone (L). PC1 is thereby ideally suited to rank individual colour elaboration along a single axis.

We used the information from the PC analysis to identify the individual with the lowest value of PC1 for each patch and species (lying furthest into the long wavelengths, see Figure 1). Note that this choice is arbitrary, we could have equally taken the individual with the highest PC1 value. We then computed ΔS from this point to all other individuals of a given patch and species to obtain a relative measure of coloration. Thus we effectively standardised all values of ΔS against the individual measurement with the lowest PC1 score. This is comparable across species and patches since it uses the same scale (all units are jnds), and higher values are associated with greater reflectance of the short wavelengths, although to a different extent, as xyz loadings on PC1 vary slightly among colour patches (Table S2 of the Online Appendix). Note that while ΔS and PC1 are highly correlated (p<0.001, r = 0.94–0.99) and conclusions are similar when using PC1 instead of ΔS to analyse seasonal colour change, PC1 was only used to identify an extreme individual in the sample to use as a reference point since it has two main shortcomings. First that the magnitude of chromatic change cannot be compared among different colour patches and second that variation in PC1 does not take into account that different cone types show different levels of signal-to- noise ratios and thus variation in coloration along certain dimensions in the avian visual space may be more difficult to perceive by the birds [47].

Note that we could have used other chromatic variables to identify extreme individuals such as carotenoid chroma for carotenoid-based patches (e.g. breast of the great tit) or UV chroma for UV-rich plumage patches (e.g. crown of the blue tit). Using these variables instead yields very similar results, which is not surprising since they are highly correlated to PC1 (Delhey et al. unpubl.data). We preferred however to use the same method for all patches especially because for some of them it is unclear which “traditional” colour variable is more suited to capture the main axis of chromatic variation (e.g. brown or grey plumage patches).

As ΔS focuses only on chromatic differences disregarding the achromatic signal (i.e. variation in brightness or luminance) we also wanted to know whether achromatic variation followed predictable patterns over the year. Most likely, in birds, achromatic variation is detected by the double cones [48], [49], and we computed the double cone quantum catch based on the sensitivity curve of the double cones of *Leiothrix lutea* [50] and formula 1 in Vorobyev et al. [40]. We then computed ΔL, the achromatic distance, from each individual (separately for each patch and species) and the individual with the lowest double cone quantum catch (i.e. the less bright individual of the sample). For ΔL we used formula 7 in Siddiqi et al. [51] with a Weber fraction of 0.05 and this variable is also measured in jnds. Higher values of ΔL then represent individuals with higher achromatic brightness relative to the darkest individual in the sample. Note that this is only true within a colour patch, as patches with higher values of ΔL are not “brighter” than patches with lower values, which simply have lower variability in brightness.

### Statistical analysis

Analysis of seasonal chromatic and achromatic changes was done separately for each species and patch. We started with the full model containing ΔS or ΔL as dependent variables, the variables month, month^2^ (to account for possible curvilinear colour changes) and the factors year and sex as main effects. As males and females could show different patterns of colour change, the interactions sex*month and sex*month^2^ were included in the full model. We then stepwise reduced the model by first removing interaction terms if not significant (i.e. p>0.05, starting with the sex*month^2^ interaction), followed by the removal of month^2^ if not significant, and always keeping month, year and sex in the final model. If either the sex*month or sex*month^2^ interactions were significant we analysed males and females separately. We did not examine the interaction between month and year due to low sample sizes and thus were unable to estimate variation in patterns between years. However, by including “year” in the model we accounted for differences in the intercept between years. As some individuals were measured more than once, bird ID was included as random factor in all models. Note however, that since repeated measurements were not forthcoming for many individuals and irregularly spread, both within and between years, it is not possible to estimate the degree of within-season consistency in coloration from this term which is included only to account for pseudo replication. Restricting the analyses only to individuals measured more than once yielded similar patterns albeit with lower power due to reduced sample sizes, suggesting that variation in population structure throughout the year played a minor role in the expression of colour change.

Additionally, for direct comparison among plumage patches, we quantified the overall change (total increase or decrease in ΔS and ΔL) over the moult year. We computed the expected difference in ΔS or ΔL (derived from the functions corresponding to the final models in Tables 1 and 2) between the first and the last month of the moult year (last month – first month) if the relationship was linear. If the best model included a quadratic term we computed two values, the expected difference between the first month and the maximum or minimum (maximum or minimum – first month), and the expected difference between the maximum or minimum and the last month (last month – maximum or minimum). If there were significant month or month^2^ by sex interactions the overall degree of change was computed separately for males and females.

**Table 1:** Statistical models describing seasonal chromatic changes (ΔS) over the year.

		Robin		blackbird						blue tit				great tit			
		breast	back	crown	bill* males	bill* females	breast*	Back males*	Back females*	crown*	cheek	breast	back	crown	cheek	breast	back
bird ID	χ^2^_1_	0.47	0.05	**10.4**	0	0.66	**19.0**	**8.13**	0.66	**12.1**	0.46	**17.8**	**57.1**	**26.9**	0	2.3	**18.3**
	p	0.50	0.80	**0.001**	1	0.41	**<0.0001**	**0.004**	0.41	**0.0005**	0.50	**<0.0001**	**<0.0001**	**<0.0001**	1	0.13	**<0.0001**
year	F	**6.31**	**5.96**	**7.3**	**12.15**	**21.2**	**10.26**	**12.9**	0.72	1.04	**19.9**	**4.17**	1.52	**13.6**	**38.03**	**7.11**	1.8
	df	**3,199.1**	**3,191**	**3,113.6**	**3,84.5**	**1,33.76**	**3,100.1**	**3,67.3**	3,31.9	3,127.6	**3,183.6**	**3,160.3**	3,112.1	**3,290.1**	**3,239.8**	**3,284.7**	3,288.1
	p	**0.0004**	**0.0007**	**0.0002**	**<0.0001**	**<0.0001**	**<0.0001**	**<0.0001**	0.54	**0.376**	**<0.0001**	0.007	**0.21**	**<0.0001**	**<0.0001**	**0.0001**	**0.145**
sex	b	**−0.735**	**−**0.34	**6.59**	-	-	**7.14**	-	-	**2.01**	**0.55**	**−1.06**	**0.805**	**3.54**	**0.36**	**−**0.19	**−0.44**
	SE	**0.24**	0.21	**0.21**	-	-	**0.262**	-	-	**0.22**	**0.12**	**0.29**	**0.15**	**0.14**	**0.084**	0.27	**0.18**
	F	**9.17**	2.51	**887.3**	-	-	**541**	-	-	**76.1**	**19.4**	**12.8**	**25.9**	**563.5**	**15.17**	0.56	**5.33**
	df	**1,142.4**	1,126.8	1,100.6	**-**	**-**	1,102.6	-	**-**	**1,130.6**	1,134.8	1,144.1	**1,144**	1,224.9	1,99.3	**1,171.8**	**1,211.4**
	p	**0.003**	0.115	**<0.0001**	-	-	**<0.0001**	-	-	**<0.0001**	**<0.0001**	**0.0005**	**<0.0001**	**<0.0001**	**0.0002**	0.45	**0.022**
month	b	**0.297**	**0.15**	**−0.101**	**−3.29**	**−1.11**	**−0.169**	**−0.13**	**−0.23**	**0.69**	**−0.22**	**−**0.014	**−0.41**	**−0.19**	**−0.19**	**−0.60**	**−0.43**
	SE	**0.042**	**0.035**	**0.05**	**0.93**	**0.24**	**0.053**	**0.045**	**0.13**	**0.24**	**0.028**	0.063	**0.14**	**0.028**	**0.021**	**0.26**	**0.17**
	F	**44.01**	**15.9**	**4.13**	**12.3**	**4.47**	**9.85**	**9.13**	**3.38**	**8.08**	**50.4**	0.04	**8.79**	**39.13**	**66.4**	**5.34**	**6.48**
	df	**1,202.9**	1,194.8	1,114.1	**1,84.9**	**3,32.3**	1,100.5	1,68	**1,33.6**	**1,152.2**	1,182.9	1,134.6	**1,128.3**	1,262.3	1,282.2	**1,257.1**	**1,237.1**
	p	**<0.0001**	**<0.0001**	**0.044**	**0.0007**	**0.01**	**0.0022**	**0.0035**	**0.074**	**0.005**	**<0.0001**	0.83	**0.0036**	**<0.0001**	**<0.0001**	**0.02**	**0.011**
month^2^	b	0.028	**−**0.0056	0.017	**0.022**	0.14	0.03	**−**0.02	0.08	**−0.056**	**−**0.001	0.012	**0.038**	**−**0.014	**−**0.005	**0.056**	**0.033**
	SE	0.014	0.014	0.017	**0.063**	0.07	0.018	0.01	0.04	**0.014**	0.014	0.021	**0.011**	0.001	0.001	**0.021**	**0.014**
	F	2.7	0.15	1.07	**11.89**	3.82	2.92	1.85	3.94	**8.81**	0.35	0.21	**11.72**	2.67	0.55	**7.17**	**6.09**
	df	1,201	1,199.5	1,112.2	**1,84.7**	1,32	1,97.2	1,59.4	1,32.2	**1,128.6**	1,184.1	1,148.6	**1,112.9**	1,234.8	1,281.3	**1,246**	**1,226.3**
	p	0.101	0.69	0.301	**0.0009**	0.059	0.09	0.178	0.055	**0.0036**	0.55	0.64	**0.0009**	0.10	0.45	**0.008**	**0.014**

Significant variables are depicted in bold. For the two studied patches where there was a significant sex*month or sex*month^2^ interaction males and females were analysed separately (sex*month^2^, bill blackbirds F_1,120.5_ = 5.9, p = 0.016; back blackbirds, F_1,115.9_ = 7.02, p = 0.0092; all others p>0.06).

*analysis carried out on Box-Cox transformed variables to improve normality of residuals. Note that here effect sizes cannot be directly compared with those of untransformed variables.

**Table 2:** Statistical models describing seasonal achromatic changes (ΔL) over the year.

		robin			blackbird					blue tit				great tit			
		breast	back males	back females	Crown males	Crown females	bill	breast	back	crown*	cheek*	breast	back	crown	cheek*	breast	back*
bird ID	χ^2^_1_	**15.8**	0	0	0	0.13	0	0	1.25	**8.72**	0.005	3.0	**7.67**	0	1.56	**10.7**	**6.8**
	p	**<0.0001**	1	1	1	0.72	1	1	0.26	**0.003**	0.94	0.08	**0.006**	1	0.21	**0.001**	**0.009**
year	F	**10.05**	0.178	**3.47**	1.38	0.624	**5.67**	1.577	**2.83**	1.18	0.78	1.77	**7.75**	2.59	**6.8**	**4.26**	**11.19**
	df	**3,227.7**	3,94.24	**3,87.42**	3,85.04	3,31.41	**3,21.4**	3,120.70	**3,124**	3,164	3,184	3,179.60	**3,180.40**	3,267.30	**3,284.30**	**3,289**	**3,288.5**
	p	**<0.0001**	0.91	**0.0194**	0.254	0.604	**0.0051**	0.198	**0.041**	0.318	0.507	0.154	**<0.0001**	0.053	**0.0002**	**0.006**	**<0.0001**
sex	b	**−**0.14	-	-	-	-	**8.00**	**−17.8**	**−9.4**	0.61	**1.02**	**0.90**	**−1.63**	**3.74**	**1.2**	**2.16**	**0.70**
	SE	0.32	-	-	-	-	**1.56**	**0.82**	**0.78**	0.59	**0.47**	**0.38**	**0.34**	**0.62**	**0.35**	**0.38**	**0.3**
	F	0.233	-	-	-	-	**26.13**	**462.23**	**143.3**	1.05	**4.71**	**5.44**	**21.53**	**36.4**	**11.55**	**31.8**	**5.4**
	df	1,175.4	-	-	-	-	**1,107.10**	**1,101.90**	**1,100.5**	1,137.3	**1,161.10**	**1,136.10**	**1,153.80**	**1,150.50**	**1,192.90**	**1,191.8**	**1,194.3**
	p	0.629	-	-	-	-	**<0.0001**	**<0.0001**	**<0.0001**	0.306	**0.031**	**0.021**	**<0.0001**	**<0.0001**	**0.0008**	**<0.0001**	**0.021**
month	b	**−0.27**	0.096	0.095	**−3.835**	0.412	1.723	**−2.238**	**−0.383**	**0.664**	**2.21**	−0.99	−0.076	**2.625**	**1.616**	−0.023	−**0.177**
	SE	**0.056**	0.078	0.099	**1.288**	0.341	0.585	**1.046**	**0.188**	**0.137**	**0.58**	0.096	0.085	**0.658**	**0.354**	0.082	**0.067**
	F	**23.08**	1.54	0.91	**8.854**	1.455	8.67	**4.578**	**4.15**	**23.29**	**14.53**	1.07	0.799	**15.9**	**20.76**	0.081	**6.84**
	df	**1,229.8**	1,101.50	1,86.79	**1,85.65**	1,32.92	1,1.963	**1,118**	1,125	**1,138.8**	**1,184.40**	1,167.60	1,169.40	**1,291.80**	**1,276.80**	1,247.4	**1,268.3**
	p	**<0.0001**	0.216	0.34	**0.0038**	0.236	0.1007	**0.034**	**0.043**	**<0.0001**	**0.0002**	0.301	0.372	**<0.0001**	**<0.0001**	0.775	**0.0094**
month^2^	b	−0.003	−0.033	0.067	**0.221**	0.0876	0.19	**0.148**	0.078	−0.022	−**0.22**	−0.03	−0.035	−**0.238**	−**0.155**	−0.026	−0.008
	SE	0.021	0.03	0.037	**0.089**	0.118	0.101	**0.072**	0.061	0.055	**0.05**	0.038	0.034	**0.052**	**0.028**	0.028	0.023
	F	0.03	1.14	3.31	**6.129**	0.544	3.51	**4.178**	1.52	0.16	**20.75**	0.57	1.05	**20.27**	**29.56**	0.858	0.134
	df	1,223.7	1,109.30	1,93.45	**1,84.52**	1,32	1,123	**1,112.80**	1,123.6	1,151.1	**1,183.70**	1,174.30	1,174.30	**1,291.30**	**1,269.90**	1,213.3	1,236.3
	p	0.854	0.287	0.072	**0.0153**	0.466	0.063	**0.0433**	0.22	0.69	**<0.0001**	0.451	0.305	**<0.0001**	**<0.0001**	0.355	0.714

Significant variables are depicted in bold. For the two studied patches where there was a significant sex*month or sex*month^2^ interaction males and females were analysed separately (sex*month^2^, back robin F_1,215.3_ = 5.06, p = 0.025; sex*month; crown blackbird, F_1,119.9_ = 7.13, p = 0.0086; all others p>0.1).

*analysis carried out on Box-Cox transformed variables to improve normality of residuals. Note that here effect sizes cannot be directly compared with those of untransformed variables.

These analyses were done with JMP 7 using linear mixed models with restricted maximum likelihood (REML). In general, residuals of the final models did not depart from normality (Shapiro-Wilk test: p>0.05). If not normal we attempted to transform the data using Box-Cox transformations. However, in some cases, the residuals of the final model still departed from normality (ΔS: robin breast, blackbird bill and great tit crown; ΔL: blackbird bill, blue tit breast and back, and great tit crown). As we were not able to achieve normality by transforming the data, these results should thus be interpreted with care.

## Results

### Patterns of chromatic change

**Figure 2:**
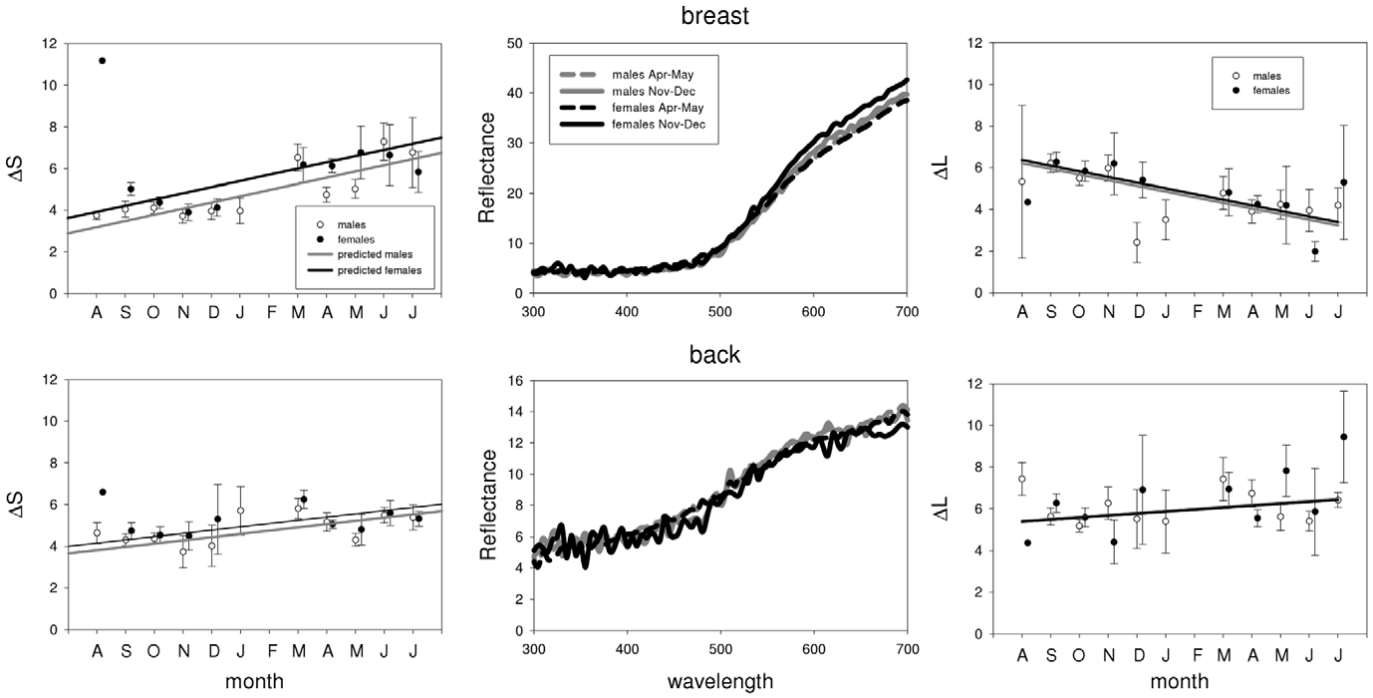
Seasonal variation in robin coloration. The left panel depicts chromatic changes (ΔS), and the right panel achromatic changes (ΔL) for males (open circles) and females (closed circles) during the year (monthly means +/− SE). Higher values of ΔS correspond to higher relative reflectance in the shorter wavelengths (UV, blue) and lower values higher relative reflectance in the longer wavelengths (red). Higher values of ΔL correspond to higher achromatic brightness relative to the darkest individual in that plumage patch. Lines for males (grey) and females (black) are derived from the final models in Tables 1 and 2. The centre panel shows average reflectance spectra for males (open symbols) and females (closed symbols) for the months of Nov-Dec (circles) and Apr-May (triangles). These months were selected because they were the first (Nov-Dec) months without moulting birds and the last (Apr-May) months before moult. Note that reflectance spectra are not to scale to highlight the differences in spectral shape.

**Figure 3:**
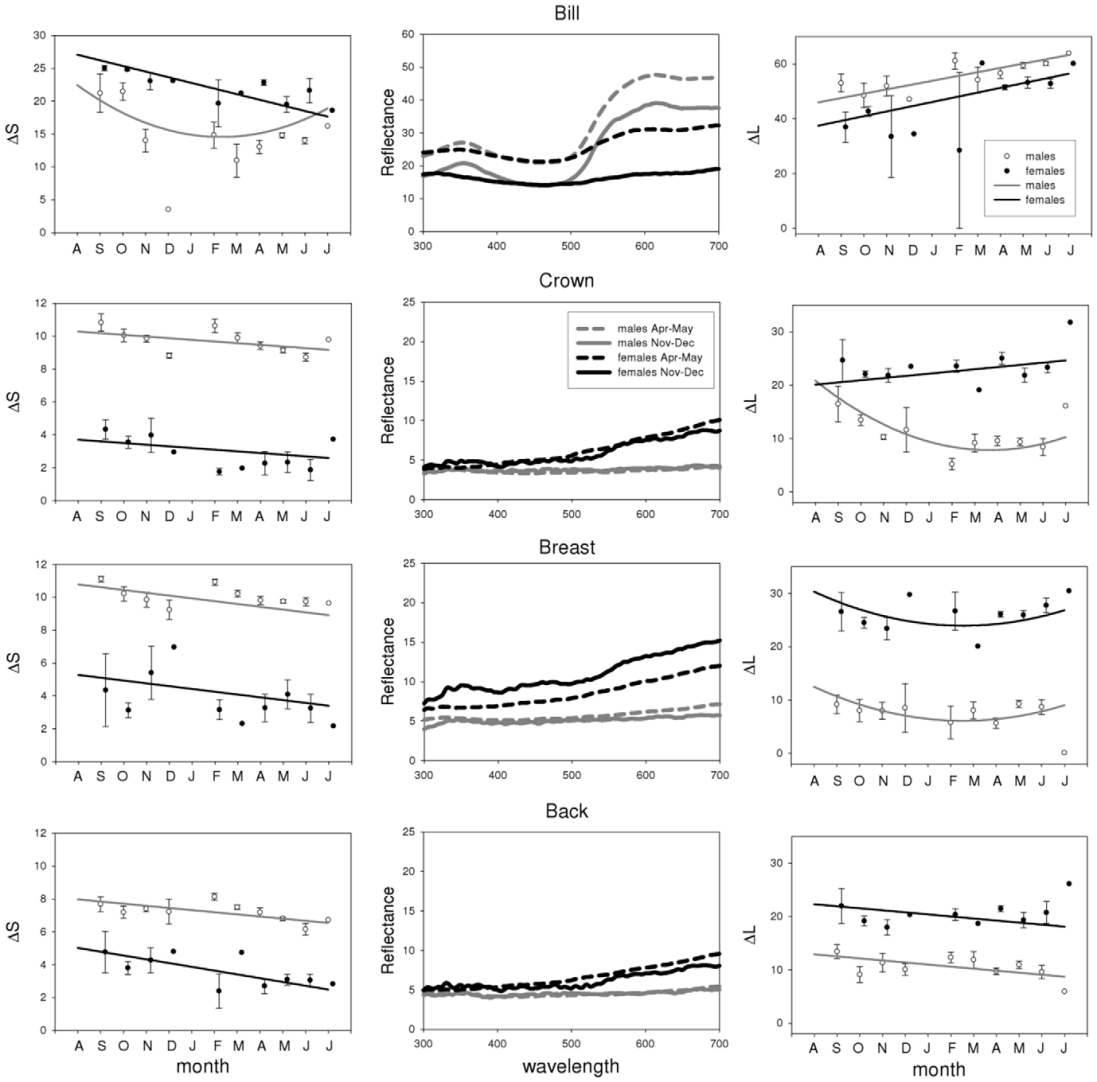
Seasonal variation in blackbird coloration. See legend of Figure 2 for more details. Note that the ΔS and ΔL graphs for the bill have not been drawn to the same scale as the plumage patches.

**Figure 4:**
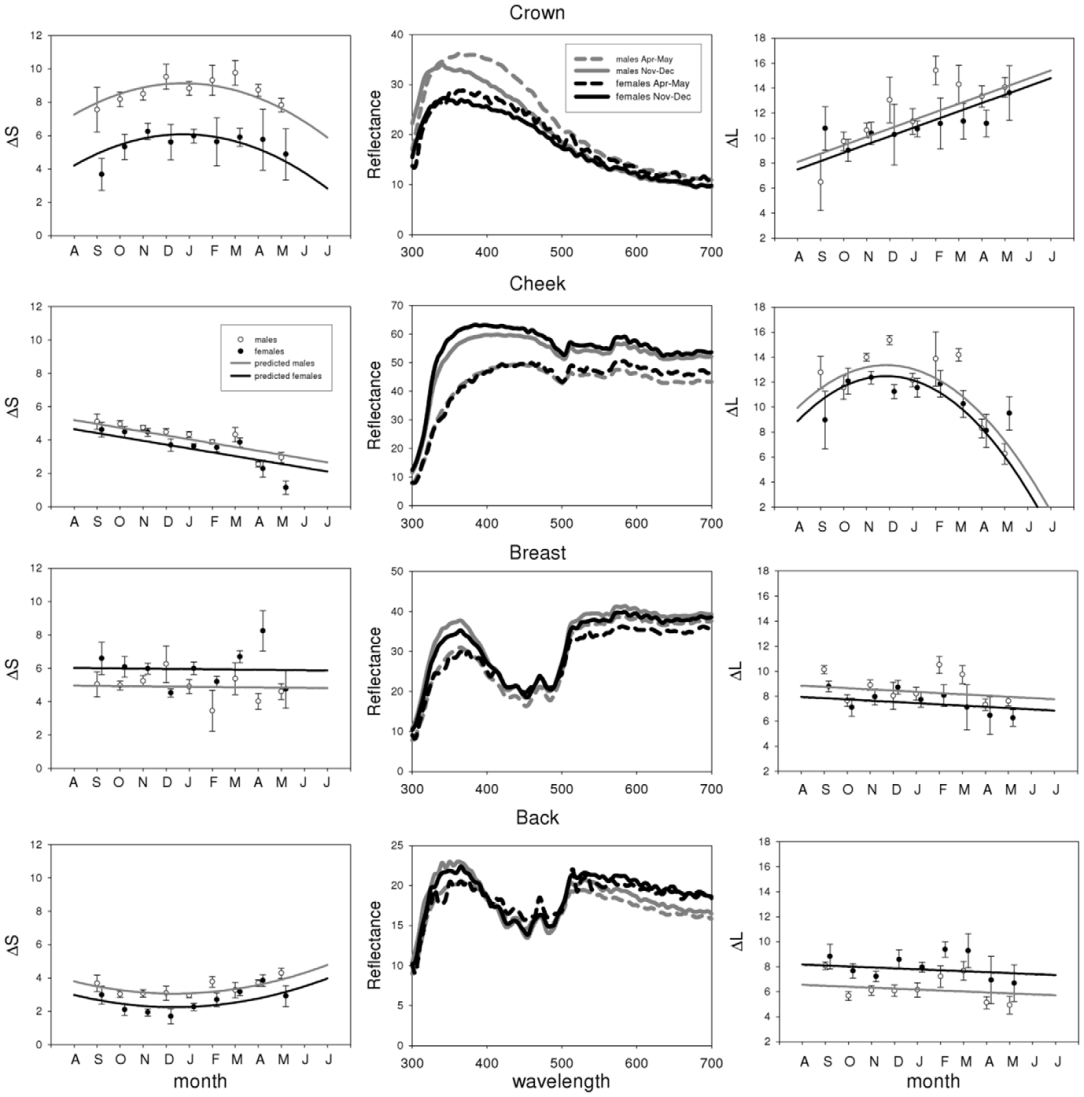
Seasonal variation in blue tit coloration. See legend of Figure 2 for more details.

**Figure 5 pone-0011582-g005:**
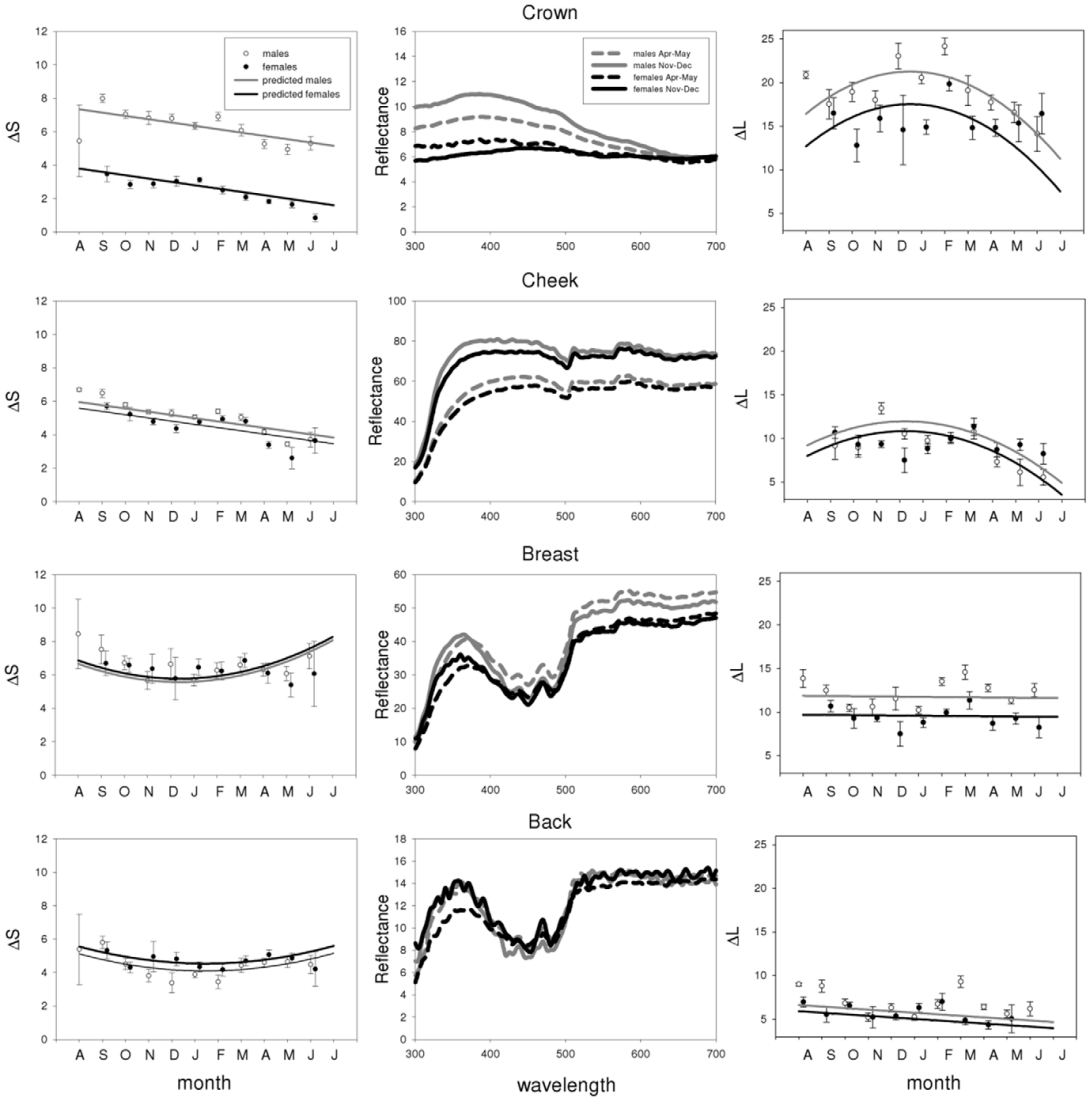
Seasonal variation in great tit coloration. See legend of Figure 2 for more details.

**Figure 6:**
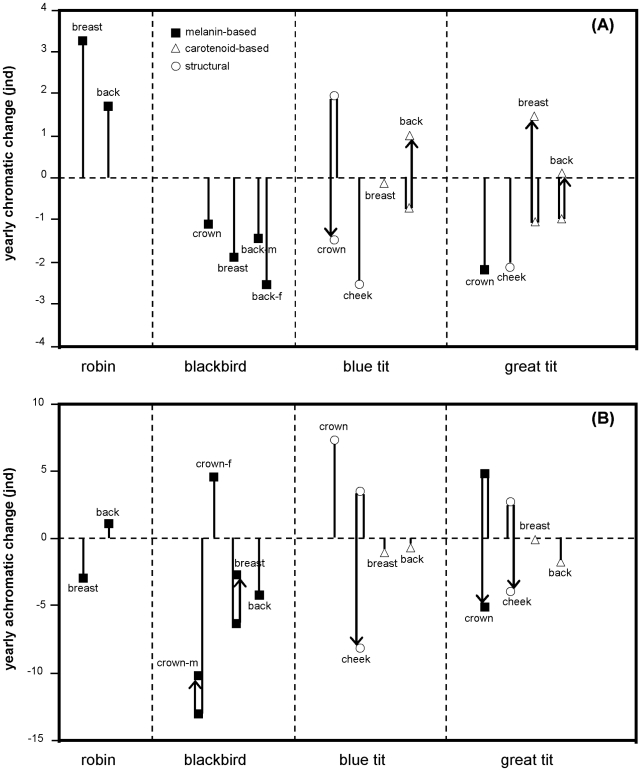
Total chromatic (A) and achromatic (B) changes over the year. Based on the final models in Tables 1 and 2 and discriminated by the main colour-producing mechanism (note that while the great tit crown has been included among melanin-based colours, its UV reflectance hints at an additional structural component, see Discussion). One value is depicted for linear changes and two (united by the arrows) for curvilinear patterns of change (the first corresponds to the minimum or maximum and the second to the final value). Positive values of chromatic changes indicate an increase in the reflectance at shorter wavelengths while positive values of achromatic changes indicate increased achromatic brightness over the year. When males and females presented different patterns of change they were depicted separately.

Plumage (and bill) coloration changed significantly over the course of the year for all species and patches, except for the breast of the blue tit (see Table 1, Figures 2, 3, 4 and 5). Many examined patches (8 out of 13) showed an overall decrease in ΔS over time, that is, later in the year these colour patches showed reduced relative reflectance at shorter wavelengths (Figure 6). However the back and breast of the robin showed a clear increase in ΔS with time, indicating increased reflectance at shorter wavelengths (Figure 2). This was also the case for the carotenoid-based back and breast plumage of the great tit and back of the blue tit (Figure 6). Two patches showed strong curvilinear patterns of colour change, the crown of the blue tit and the bill of the blackbird. The crown colour of the blue tit showed the highest values of ΔS (high relative reflectance of shorter wavelengths, Figure 4) in winter, while the bill of the blackbird showed the lowest values of ΔS at the same time (high relative reflectance of longer wavelengths, Figure 3). Curvilinear patterns were not as strong in the other patches with significant month^2^ effects (back of the blue tit, back and breast of the great tit and back of female blackbirds, Table 1, Figures 3, 4, 5). Absolute levels of plumage chromatic change (the sum of the absolute value of all chromatic changes over the year, computed from Figure 6A) were on average slightly higher for structural colours (3.3 jnd, range  = 2.1–5.4, n = 3) than for carotenoid-based (2 jnd, range  = 0.2–3.6, n = 4) and melanin-based colours (2 jnd, range  = 1.1–3.2, n = 7).

Most colour patches were sexually dichromatic: males and females differed significantly from each other in values of ΔS in all but two cases (breast of the great tit and back of the robin). Significantly different patterns of change in ΔS between the sexes (i.e. a significant interaction term between month and sex) were present only for the coloration of back and bill of the blackbird (Table 1, Figure 3).

### Patterns of achromatic change

Significant seasonal changes in achromatic brightness (ΔL) were detected in 9 of our 14 plumage patches (Table 2, Figures 2, 3, 4 and 5). In five cases brightness had increased by the end of the moult year whereas decreases were detected in the remainder nine, although for some of these the change was only slight and non-significant (e.g. back and breast of blue tit and great tit, Figure 6B). Curvilinear patterns of change were evident for five plumage patches. Absolute levels of achromatic change in plumage over the year (the sum of the absolute value of all achromatic changes over the year, computed from Figure 6B) were highest for structural colours (mean  = 10.9 jnd, range  = 7.3–15.5, n = 3), intermediate for melanin-based colours (mean  = 6.6 jnd, range = 1–15.4, n = 8) and lowest for carotenoid-based colours (mean  = 1 jnd, range  = 0.2–1.9, n = 4). Sexual dichromatism in ΔL was found in most plumage patches except for the breast and back of the robin and the crown of the blue tit. Differences between the sexes in the pattern of change (i.e. significant month*sex or month^2^*sex interactions) were only found for the back of the robin and the blackbird crown (Table 2).

## Discussion

All but one of the studied colour patches (the breast of the blue tit) showed significant chromatic changes and nine out of fourteen significant achromatic changes over the year. This suggests that seasonal changes in coloration independent of moult may be widespread among different bird species and colour types.

### Differences among colour types

#### Chromatic variation

We investigated plumage patches which differed in the main mechanism of colour production, distinguishing structural, carotenoid- and melanin-based colours. Since varying proportions of structural as well as carotenoid- and/or melanin-based components often contribute to the final reflectance spectrum [52], [53] and only little is known about the exact contributions, this subdivision is not absolute, but rather reflects the dominant colour producing mechanism of a given patch.

Despite the fact that melanin-based coloration has been hypothesised to show reduced levels of seasonal variation [19] we found that all patches of melanin-based colours changed seasonally. Melanins are the most common feather pigments, they are produced in the feather follicle and confer several benefits such as mechanical strength, resistance to bacterial degradation and protection from UV light and oxidative stress [25]. Melanised feathers are often assumed to be poor indicators of individual phenotypic quality and less sensitive to abiotic and biotic environmental influences ([27], [30] but see [28], [54]). Previous studies show that melanin-based colours showed either decreases in saturation (i.e. feathers of the brownish back in two species of tit (*Parus montanus* and *P. palustris*) became greyer [16]) or no seasonal change as in the black crown of the great tit [19]. In contrast to the latter study, our analysis identified significant seasonal changes in the black crown colour of great tits. The melanin-based crown of the great tit shows relatively high reflectance in the UV (especially in males), indicative of a structural component (Figure 5, see also [55]). The decrease in ΔS was mainly due to a decrease in those short wavelengths (Figure 5). Figuerola & Senar [19] investigated colours in the human visible spectrum only and therefore might have missed the changes occurring in the UV. The black and dark-brown predominantly melanin-based colour patches in the blackbird followed roughly the same pattern as the great tit crown with a decrease in relative reflectance at shorter wavelengths (Figure 3) while the brown back and particularly the red breast of the robin showed changes in the opposite direction (Figure 6). This was due to a decline in long wavelength reflectance (between 550 and 700 nm, Figure 2) and presumably corresponds to reduced “redness” of the breast patch. The reason behind this pattern is still unknown, but may be related to the fact that phaeomelanins instead of eumelanins are the dominant pigment in red plumage. The two forms of melanin are biochemically distinct, and differently sensitive to internal physiology (sex hormones, oxidative stress, toxic metal concentrations; [25]. Whether phaeomelanins are more sensitive to abrasion or the influence of UV radiation than eumelanins is unclear and deserves further study.

Carotenoid-based colours have been shown to be sensitive to a variety of environmental influences after moult such as UV-radiation or bacterial degradation [11], [17], [18], [54], [21] and we therefore expected large changes to occur over time. We found significant chromatic changes for all carotenoid-containing patches, except for the blue tit breast, but overall changes over the year were not necessarily larger than for other colours (Figure 6, excluding the blackbird bill, see below). This was at least partly due to the curvilinear nature of coloration change over the year, with an initial decrease in ΔS followed by an increase towards spring (Figures 4, 5). The initial reduction of UV reflectance is presumably due to dirt accumulation, given that dirt affects UV and short wavelengths relatively more [5], [13]. Subsequent increase in relative short wave reflectance could result from photo bleaching of carotenoids [12] since yellow carotenoids fading from the plumage can reveal underlying structural coloration, and thus cause a shift towards increased reflectance at shorter wavelengths later in the year (for an interesting example see [17]).

Clearly different from the carotenoid-based plumage colours studied here, the bill of the blackbird showed the strongest changes in colour expression over the year of all studied patches (compare the scale of colour variation in Figure 3). Carotenoid-based soft parts in general are highly dynamic traits that have been shown to respond rapidly to a variety of physiological and social factors in a number of species (e.g. [56]–[58]). Likewise, coloration of the bill of the blackbird changes with individual condition [37] nutritional access to pigments or exposure to parasites [59]. Our study confirms the expectation that such dynamic traits also show extensive seasonal variation that is consistent at a population level.

Structural colours differ from pure pigment-based colours in their mechanisms of development and physical composition [26], which probably affects temporal changes in colour expression, especially the fact that they often present high relative reflectance at short wavelengths. We found strong linear declines in ΔS throughout the year for the structure-based white coloration of the cheek patches (Figures 4, 5, 6), while the UV/blue crown of the blue tit showed a characteristic negative quadratic pattern. The seasonal pattern of colour variation found for this patch is very similar to that described by Örnborg et al. [5] and Delhey et al. [22], who both found an increase in relative UV reflectance between post-moult and late winter followed by a subsequent decrease. The initial increase in ΔS in the UV-reflecting blue tit crown could result from special properties of the crown feathers, where the UV-reflecting nanostructure of the barbs is revealed by rapid abrasion of the smaller melanised barbules, while the breakage of barbs and accumulation of dirt and fat (that strongly absorb UV wavelengths) on the feathers probably leads to the observed shift towards longer wavelengths later on [5]. The initial increase could be an adaptation to maximise the colour signal just before the breeding season when new pairs are formed [60]. It is interesting that the UV-rich crown coloration of the related great tit does not show this initial increase in ΔS, possibly due to the presumed larger melanin content which may strengthen the black crown feathers. In the future it might be worthwhile to relate feather structure, patterns of abrasion and seasonal colour change over the year for different types of structurally coloured feathers to confirm or not whether they are more prone to seasonal changes as our data seems to suggest.

#### Achromatic variation

Changes over the moult year in perceived luminance or brightness were also widespread in our sample. Patterns of change varied considerably even within the same type of colour producing mechanism and it is difficult to find general patterns. This is best seen among melanin-based colours where either increases, decreases or both, could be observed. As discussed above these patterns do not agree with the hypothesis that melanin-based colours should be especially resistant to abrasion or damage. Also against expectations, carotenoid-based plumage patches showed consistently very small changes in achromatic brightness over the year (Figure 6). Finally, among structural colours white cheek patches in both tit species became less bright at the end of the year after an initial increase with a winter maximum (Figures 4–6). While the initial increase is puzzling, the subsequent decline in achromatic brightness may be due to the accumulation of dirt on the white feathers. The other structural colour in our sample, the crown plumage of the blue tit, showed a linear increase in achromatic brightness over the year. This is in agreement with other studies [5], [22] and may be due to the action of keratinolytic bacteria that reduce the thickness of the light-absorbing keratin cortex exposing the colour-producing spongy layer in the feather barbs [3].

### Differences between males and females

We predicted that patterns of colour change might differ between males and females, at least regarding known sexually selected colours, which presumably are under strong selection in males, but less so in females. Birds have been shown to actively modify the coloration of their plumage [6], [9], so males might be able to enhance signalling properties of sexually selected traits and this should lead to different patterns between males and females. Contrary to our expectations, we found significant sex differences in chromatic change only for the back and the bill of the blackbird, and different patterns of achromatic change only for the crown of the blackbird (even though we found a significant sex*month interaction for the robin back as well, differences between males and females were very small, Table 2, Figures 2 and 6). For the carotenoid-based bill colour, we found a strong decrease in ΔS in the first half of the year in males, but less in females. This means that bill coloration of males became more orange/red, i.e. more intensely coloured, probably due to increased deposition of carotenoid pigments [61]. Male bill colour is presumably sexually-selected [59] and accordingly, bill colour of males showed maximum expression at the end of winter (Figure 3), the time when new pairs are formed [62]. The back of the blackbird is not known to be a sexually selected trait and the causes of sexual differences in the patterns of colour change remain unclear. Note however that differences between males and females are by far not as dramatic as for the bill (Figure 3). Differences between the sexes in achromatic change over the year for the blackbird crown are also difficult to explain. On the other hand, the lack of sexual differences in colour change for most of the studied patches, including known sexually selected traits such as the crown of the blue tit, suggests that individual birds are largely unable to prevent or delay the deterioration of plumage coloration and the seasonal changes we uncovered are most likely a result of passive processes (but see [63]).

### Conclusions

Our results show that carotenoid- and melanin-based pigmentary colours, as well as (predominantly) structural colours, can change significantly over the year, although patterns differ considerably both within and between colour types. Our limited sample suggests that structural colours may be particularly susceptible to seasonal colour changes but this needs to be confirmed with larger sample sizes. However, the important question remains, do these changes affect colour signalling? In general, overall changes over the year were of a magnitude that should be discriminable for birds (i.e. in most cases yearly changes exceeded the theoretical discrimination threshold of 1 jnd, see Figure 6). Note however, that the exact value of this threshold depends on the sensitivity functions of the single cones and their abundance in the retina. These parameters are currently known only for relatively few species. Thus, while the average parameter values we use are close to those of some of our study species (blue tit, blackbird, [44]) this may not necessarily be the case for the other two. This uncertainty may affect the exact values computed by the models but is unlikely to change the general patterns of seasonal changes (see also [40]).

To what extent seasonal changes could affect signalling will depend on whether signalling is important year-round or only at certain times. For example, while pairing in the blue tit may take place early in the year, at the peak of colour expression, variation in male crown colour has been shown to affect female reproductive decisions as late as the chick feeding period [64], [65] when the plumage is considerably faded (Figure 4). Thus, the date of colour measurement may have important consequences for the outcome of studies into signalling functions of avian plumage. Neglecting seasonal change might result in measuring variation in coloration which does not reflect intrinsic variation due to pigmentation and/or feather structure. This is of particular concern if coloration is not measured at the time that signalling takes place (for example if we measure coloration at the end of the breeding season, e.g. [66], [67]). This last problem may be somewhat mitigated if individual coloration is correlated within a season [22], an assumption that should be confirmed for each studied plumage patch. We emphasize that there is a need for studies investigating annual changes in plumage and soft-part coloration for a broader variety of species, particularly expanding the comparison of colours based on different mechanisms, for example including plumage colours pigmented with red in addition to yellow carotenoids, more eumelanin-based traits and more structural colours (for example iridescent colours). Moreover, further studies should focus on the patterns of within-individual changes in coloration, particularly individual consistency of relative colour signalling and function and/or consequences of individual changes. Meanwhile, our results show that visual modelling now makes it possible to perform meaningful comparisons of colour variation over the year between different types of colours.

## Supporting Information

Table S1 Sample sizes for each study species, discriminated by sex and month.
(0.01 MB PDF)

Table S2 Statistical details on the Principal Component Analysis. Only results for PC1 are presented.
(0.01 MB PDF)
