# Supplementary material for: Seasonal Changes in Colour: A Comparison of Structural, Melanin- and Carotenoid-Based Plumage Colours
Source: PLoS One. 2010 Jul 14;5(7):e11582. doi: 10.1371/journal.pone.0011582 (PMC2904367; doi:10.1371/journal.pone.0011582)
Supplement: Table S1 — Sample sizes for each study species, discriminated by sex and month. (0.01 MB PDF) [file pone.0011582.s001.pdf]

Robin
Breast

month	Males	Females
August	2	1
September	25	21
October	38	24
November	8	6
December	5	2
January	3	0
February	0	0
March	13	8
April	18	29
May	11	4
June	7	4
July	4	3

Robin
Back

month	Males	Females
August	2	1
September	25	21
October	38	24
November	8	6
December	5	2
January	3	0
February	0	0
March	13	8
April	18	29
May	11	4
June	7	4
July	4	3

Blackbird
Crown

month	Males	Females
August	0	0
September	3	2
October	8	13
November	3	3
December	2	1
January	0	0
February	4	2
March	8	1
April	17	4
May	39	7
June	7	4
July	1	1

Blackbird
Bill

month	Males	Females
August	0	0
September	3	3
October	8	13
November	3	3
December	1	1
January	0	0
February	4	2
March	8	1
April	17	4
May	39	7
June	7	4
July	1	1

Blackbird
Breast

month	Males	Females
August	0	0
September	3	3
October	8	13
November	3	3
December	2	1
January	0	0
February	4	2
March	8	1
April	17	4
May	39	7
June	7	4
July	1	1

Blackbird
Back

month	Males	Females
August	0	0
September	3	3
October	8	13
November	3	3
December	2	1
January	0	0
February	4	2
March	8	1
April	17	4
May	39	7
June	7	4
July	1	1

Table S1 (cont.)

Blue tit
Crown

month	Males	Females
August	0	0
September	5	4
October	17	10
November	25	15
December	3	4
January	19	20
February	3	4
March	7	9
April	27	4
May	15	3
June	0	0
July	0	0

Blue tit
Cheek

month	Males	Females
August	0	0
September	5	4
October	17	10
November	25	14
December	3	4
January	19	20
February	3	4
March	6	9
April	27	4
May	15	3
June	0	0
July	0	0

Blue tit
Breast

month	Males	Females
August	0	0
September	5	4
October	17	10
November	25	15
December	3	4
January	19	20
February	3	4
March	7	9
April	27	4
May	15	3
June	0	0
July	0	0

Blue tit
Back

month	Males	Females
August	0	0
September	5	4
October	17	10
November	25	15
December	3	4
January	19	20
February	3	4
March	7	9
April	27	4
May	15	3
June	0	0
July	0	0

Great tit
Crown

month	Males	Females
August	2	0
September	6	6
October	18	8
November	15	5
December	6	4
January	37	22
February	15	15
March	17	18
April	38	24
May	27	7
June	7	2
July	0	0

Great tit
Cheek

month	Males	Females
August	2	0
September	6	6
October	18	8
November	15	5
December	6	4
January	37	22
February	15	15
March	17	18
April	38	24
May	27	7
June	7	2
July	0	0

Great tit
Breast

month	Males	Females
August	2	0
September	6	6
October	18	8
November	16	5
December	6	4
January	37	22
February	15	15
March	17	18
April	38	24
May	27	7
June	7	2
July	0	0

Great tit
Back

month	Males	Females
August	2	0
September	6	6
October	18	8
November	16	5
December	6	4
January	37	22
February	15	15
March	17	18
April	38	24
May	27	7
June	7	2
July	0	0
