# Supplementary material for: Seasonal Changes in Colour: A Comparison of Structural, Melanin- and Carotenoid-Based Plumage Colours
Source: PLoS One. 2010 Jul 14;5(7):e11582. doi: 10.1371/journal.pone.0011582 (PMC2904367; doi:10.1371/journal.pone.0011582)
Supplement: Table S2 — Statistical details on the Principal Component Analysis. Only results for PC1 are presented. (0.01 MB PDF) [file pone.0011582.s002.pdf]

	Robin (*Erithacus rubecula*)	
PC1	breast	back
% variance explained (rescaled)	94.930949	92.1345563
Eigenvector x (rescaled)	-0.98154347	-0.77933832
Eigenvector y (rescaled)	0.9680206	0.99573554
Eigenvector z (rescaled)	0.87270378	0.77912564

	Blackbird (*Turdus merula*)			
PC1	breast	back	bill	crown
% variance explained (rescaled)	97.1967981	90.9287661	96.6531695	94.726777
Eigenvector x (rescaled)	-0.96369067	-0.88875305	-0.90645623	-0.93133805
Eigenvector y (rescaled)	0.99562986	0.98257205	0.99762345	0.99004834
Eigenvector z (rescaled)	0.9745006	0.94100324	0.96117288	0.96980034

	Blue tit (*Cyanistes caeruleus*)			
PC1	breast	back	cheek	crown
% variance explained (rescaled)	97.1497801	85.4253795	86.5634923	95.0965088
Eigenvector x (rescaled)	-0.49455575	-0.28072823	-0.65493958	-0.82929662
Eigenvector y (rescaled)	0.99967874	0.99847224	0.98662539	0.99860827
Eigenvector z (rescaled)	0.75639949	0.45699887	0.82879049	0.93862098

	Great tit (*Parus major*)			
PC1	breast	back	cheek	crown
% variance explained (rescaled)	97.1437741	92.4541986	88.8771527	94.0041606
Eigenvector x (rescaled)	-0.66300193	0.05118706	-0.74802687	-0.86532031
Eigenvector y (rescaled)	0.99960731	0.99983943	0.9871832	0.9966362
Eigenvector z (rescaled)	0.76597844	0.53995001	0.83858321	0.93734569
